# Recent progress in the effect of ferroptosis of HSCs on the development of liver fibrosis

**DOI:** 10.3389/fmolb.2023.1258870

**Published:** 2023-09-26

**Authors:** Rui Tang, Jing Luo, Xiaoxia Zhu, Pengyu Miao, Hong Tang, Yue Jian, Sibei Ruan, Feng Ling, Mingxi Tang

**Affiliations:** ^1^ School of Basic Medical Sciences, Southwest Medical University, Luzhou, Sichuan, China; ^2^ Department of Pathology, Affiliated Hospital of Southwest Medical University, Luzhou, Sichuan, China

**Keywords:** ferroptosis, liver fibrosis, HSCs, iron ions, lipid peroxidation

## Abstract

Fibrosis is a common pathological process that must take place for multiple chronic liver diseases to develop into cirrhosis and liver cancer. Liver fibrosis (LF) is regulated by various cytokines and signaling pathways in its occurrence and development. Ferroptosis is an important mode of cell death caused by iron-dependent oxidative damage and is regulated by iron metabolism and lipid peroxidation signaling pathways. In recent years, numerous studies have shown that ferroptosis is closely related to LF. As the main material secreted by the extracellular matrix, hepatic stellate cells (HSCs) are a general concern in the development of LF. Therefore, targeting HSC ferroptosis against LF is crucial. This review describes the current status of treating LF by inducing HSC ferroptosis that would aid studies in better understanding the current knowledge on ferroptosis in HSCs and the future research direction in this field.

## 1 Introduction

The liver is an essential digestive organ for the human body. If its function is impaired, it will affect the normal metabolism of the human body (Roehlen et al., 2020). Liver fibrosis (LF) is a pathological phenomenon that causes the accumulation of the extracellular matrix (ECM) due to persistent and repeated liver damage (Bertheloot et al., 2021). It is mainly characterized by the excessive accumulation of the ECM (such as collagen, fibronectin, and laminin) in the liver, triggering a persistent wound-healing response associated with various injuries (such as chronic viral and chemical hepatitis, genetic and metabolic diseases, and autoimmune diseases) (Wu and Zern, 2000; Iredale, 2003). Hepatic stellate cells (HSCs) play a crucial role in the development, progression, and reversal of hepatic fibrosis (Trivedi et al., 2021; Park et al., 2019). HSCs are also called vitamin A storage cells, lipid cells, stromal cells, fat storage cells, or Ito cells (Senoo et al., 2010). Under normal conditions, HSCs are mainly distributed around the hepatic sinusoids, representing approximately 8%–14% of the hepatocytes (Kumar et al., 2018), and are placed in a dormant state (Garbuzenko, 2022). The main function of HSCs is to store vitamin A and fat and to provide energy reserve and metabolic functions for the liver (Hong et al., 2018). However, under chronic liver injury and inflammation (Li et al., 2021; Heymann et al., 2016), HSCs are activated and change from dormant to active fibroblasts (Trivedi et al., 2021). When the liver is damaged, the HSC phenotype changes, or they are converted into fibroblasts by activating α-smooth muscle actin (Trivedi et al., 2021), promoting the secretion of collagen (Khomich et al., 2019). Fibroblasts secrete matrices, such as smooth muscle actin, type I and III collagen fibers, fibronectin, thrombin-sensitive protein-1, and proteoglycans, and produce lysyl oxidase (LOX), lysyl oxidase-like protein (LOXL), and transglutaminase with HSCs to mediate the cross-linking of collagen (Cannito et al., 2017; Schnabel et al., 2004; Perepelyuk et al., 2013; Vallet et al., 2019). When the injury stimuli persist, HSCs are continuously activated and transform into fibroblasts, which destroy the balance of the liver sinus, leading to excessive accumulation and deposition of the ECM in the Disse space. When the liver is damaged, changes in the HSC phenotype aggravate liver fibrosis, leading to the formation of liver scar tissue (Lackner et al., 2019). The progression of fibrosis seriously affects liver function and may eventually lead to cirrhosis, liver failure, and even the development of liver cancer (Chen et al., 2020b).

**FIGURE 1:**
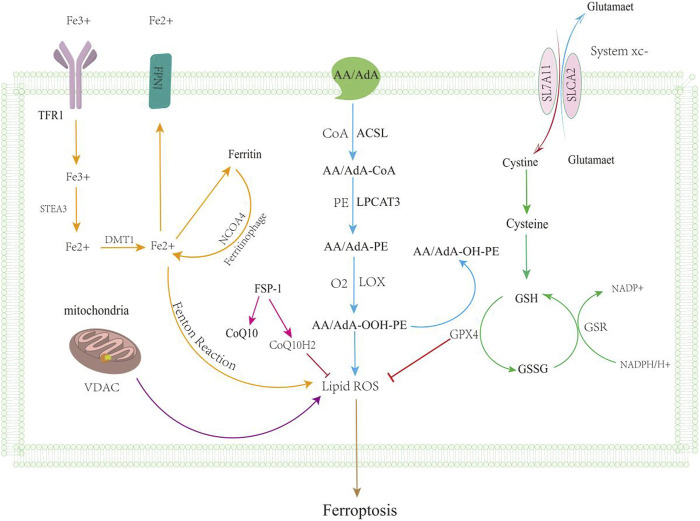
Ferroptosis mechanisms. Ferroptosis is mainly caused by the iron metabolic pathway (orange line), GSH function (green line), lipid peroxidation (blue line), and VDAC pathway (purple line); regulation of ferroptosis by the ferroxidase inhibitory protein 1-coenzyme Q10 is shown by the pink line. After iron overload, free Fe2 + can form hydroxyl radicals through the Fenton reaction, promoting lipid ROS leading to ferroptosis, and excessive iron accumulation can also lead to ferroptosis. Massive exploitation of VDAC channels leads to mitochondrial hyperpolarization, increased ROS accumulation, ferroptosis. GSH levels reduced, leading to reduced antioxidant levels; it can lead to the accumulation of reactive oxygen species. Lipid metabolism leads to lipid peroxidation through a series of reactions.

In recent years, ferroptosis has received much attention in the medical field as a new form of cell death. Ferroptosis is an iron ion-dependent form of cell death, which includes the accumulation of iron ions, the accumulation of ROS, and lipid peroxidation (Chen et al., 2020a; Pan et al., 2021) (Figure 1). Iron plays a key role in ferroptosis and promotes oxygen-free radicals and lipid peroxidation. Iron overload is an important factor in ferroptosis, and intracellular iron regulatory proteins regulate intracellular iron content (Chen et al., 2023). Iron-based autophagy is stable by regulating intracellular iron content (Hou et al., 2016). Excessive phagocytosis of ferritin leads to cell death and the onset of disease. NCOA4 associates with ferritin and regulates the phagocytic process of ferritin. The downregulation of ATG5 and ATG7 was able to inhibit ferroptosis, reduce the levels of free irons and lipid peroxides, and increase the content of glutathione (Hou et al., 2016). Iron content is associated with the occurrence of ferroptosis, and the inhibition of autophagy and the decreased expression of the NCOA4 gene induce the development of ferroptosis (Hou et al., 2016; Mancias et al., 2015). Autophagy-related genes are also factors that regulate the development of ferroptosis (Gao et al., 2016). The injury of lipid peroxidation is one of the causes of ferroptosis and is triggered by oxidative stress. ROS attacks on biofilms cause damage, but the normal liver has an antioxidant system to consume reactive oxygen species. ACSL4 catalyzes the conversion of long-chain fatty acids into lipid acyl-coenzymes and plays a key role in oxidative stress (Yuan et al., 2016). AA and AdA and A-CoA and AdA-CoA esteracetyl-CoA, forming AA/AdA-PE, lead to the destruction of membrane structure and promote ferroptosis (Valerian et al., 2016; Doll et al., 2017; Kuang et al., 2020). The inhibition of system xc reduces GSH levels and affects the function of GPX 4 (Du et al., 2022). GPX 4 is a key regulator of regulating ferroptosis for resolving lipid peroxides (Brigelius-Flohe et al., 2013). The FSP1-CoQ10-NAD(P)H pathway, as an independent parallel system, cooperates to prevent phospholipid peroxidation and ferroptosis of GSH-GPX4 (Bersuker et al., 2019; Doll et al., 2019). The GCH1/BH4/DHFR pathway and DHODH pathway are also key pathways for resistance to ferroptosis (Kraft et al., 2020; Mao et al., 2021). Nrf 2 is a transcription factor regulating the redox balance that suppresses the development of ferroptosis (Tsai et al., 2020; Sun et al., 2016). The p62-Keap1-Nrf 2 pathway was also able to inhibit the development of ferroptosis (Tsai et al., 2020; Sun et al., 2016; Sun et al., 2020). Elevated iron levels are a common feature of all these profibrotic diseases, suggesting that iron load may aggravate the degree of LF and promote disease progression. Excessive intracellular Fe accumulation can produce hydroxyl groups and peroxy radicals through oxidation reactions, promote polylipid peroxidation, and eventually lead to ferroptosis (Gaschler and Stockwell, 2017). In addition, HSCs are also an important part of LF, so ferroptosis plays an important role in hepatic stellate cell activation and LF progression. Understanding the mechanisms of ferroptosis that target HSC death and developing targeted therapeutic approaches have great potential for improving the therapeutic efficacy of LF.

This review summarizes the pathogenesis and progression of ferroptosis in LF, explores the specific mechanism of action of HSCs in ferroptosis, summarizes the role of HSCs in intervention with LF through the ferroptosis pathway, and illustrates the effectiveness of treating ferroptosis in LF through HSCs. Therefore, our review aims to reveal the pathways and targets of HSCs targeting ferroptosis to treat liver fibrosis, thus allowing researchers to have a more complete understanding of HSCs and providing another insight by targeting HSC ferroptosis to treat liver fibrosis.

## 2 HSCs and ferroptosis in liver fibrosis

### 2.1 Ferroptosis in liver fibrosis

Since the first study reported the role of ferroptosis in LF (Sui et al., 2018), evidence of ferroptosis in the pathogenesis of LF has increased. In recent years, many clinical studies have found that severe pathological iron overload symptoms are often observed in LF patients, suggesting that pathological iron overload may play a critical role in the progression of LF. Yang et al. (2020) observed a significant increase in Fe2 + and also in the expression of GPX 4, TRF, and SLC7A11 proteins in the kidneys of fibrotic mice. TRF deficiency is an autosomal recessive metabolic disorder with low expression of TRF associated with severe anemia, hepatic iron overload, and fibrosis. Yu et al. (2020) reported that mice fed a high-iron diet showed iron accumulation in multiple organs with liver injury and LF. However, the involvement of SLC39A14 genes in TRF knockout mice, intracellular Fe2 + transport in the liver, and TRF-specific TRF and SLC39A14 double knockout mice demonstrate that hepatic TRF deficiency could induce cellular ferroptosis to induce LF. However, the involvement of the SLC39A14 gene in intracellular Fe2 + transport was further clarified in TRF knockout mice and in TRF-specific TRF and SLC39A14 double knockout mice, demonstrating that hepatic TRF deficiency induced ferroptosis to induce LF. Solute carrier family 39 member 14 (SLC39A14) is a key protein that causes non-transferrin-bound iron accumulation in hepatocytes in the absence of transferrin, and knockdown of SLC39A14 is able to completely reverse the LF caused by non-transferrin-bound iron deposition in hepatocytes. In the Bama mini-pig model of excessive iron-induced myocardial fibrosis, the amount of iron deposition was positively correlated with the degree of myocardial fibrosis (Yu et al., 2020). Solute carrier family 39 member 14 (SLC39A14) is a key protein causing non-transferrin-bound iron accumulation in hepatocytes in the absence of transferrin, and knockdown of SLC39A14 is able to completely reverse LF due to non-transferrin-bound iron deposition in hepatocytes.

### 2.2 Ferroptosis in HSCs

A number of basic studies have shown that static HSCs do not express the transferrin receptor, while activated HSCs overexpress the transferrin receptor (Karvar et al., 2020). The transferrin receptor has a strong affinity for extracellular free iron and transports iron into activated HSCs via the endocytic pathway. Activated HSC intracellular iron can produce large amounts of free radicals through the Fenton response and induce the expression of inflammatory cytokines. These stimulators can continuously activate HSCs through autocrine or paracrine pathways. This pathological circulation may lead to a substantial accumulation of iron in the activated HSCs (Bridle et al., 2003; Ruddell et al., 2009; Yang et al., 2020). ELAV-like RNA-binding protein 1 (ELAVL1) (Zhang et al., 2018), bromodomain-containing protein 7 (BRD7) (Zhang et al., 2020b), ZFP36 ring finger protein (ZFP36) (Zhang et al., 2020a), and tripartite motif-containing protein 26 (TRIM26) (Zhu et al., 2021a) have been implicated in regulating ferroptosis of HSCs. Embryonic lethal abnormal vision, such as 1/human antigen R (ELAVL1/HuR), also plays a key role in promoting ferroptosis in LF cells. The upregulation of ELAVL1/HuR can induce ferroptosis in hematopoietic stem cells by stabilizing the expression of autophagy-related protein Beclin-1, promote the degradation of TRF, and release Fe2 +, leading to the imbalance of iron metabolism (Zhang et al., 2018). BRD7 has the ability to act as a ferroptosis inducer targeting HSCs (Zhang et al., 2020b). Another study showed that direct binding of BRD7 promotes p53 mitochondrial translocation, subsequently forming a complex with solute carrier family member 2,528 (SLC25A28) and enhancing its activity, causing excessive deposition of p53 mitochondrial translocation. Zhang et al. (2020b) further reported a critical role of the BRD7-p53-SLC25A28 axis in regulating ferroptosis in HSCs, with system Xc-inhibition, GPX4 inhibition, and GSH depletion mediated by BRD7 upregulation triggerring p53 mitochondrial translocation through direct binding to the N-terminal trans-activated structural domain, which exacerbates mitochondrial iron accumulation, electron transport chain hyperfunction, lipid peroxidation, and ultimately, iron-dependent ferroptosis. Zhang et al. (2020a) showed that the RNA-binding protein ZFP36 prevents ferroptosis by regulating the autophagy signaling pathway in HSCs. ZFP36 is an RNA-binding protein that disrupts autophagy-related 16-like 1 (ATG16L1) mRNA, thereby suppresses macroautophagy/autophagy activation and mediates ferroptosis resistance. TRIM26 is downregulated in the fibrotic tissue of liver tissue, and the overexpression of TRIM26 accelerates the degradation of lipid proteins by mediating SLC7A11 ubiquitination, promotes lipid peroxidation accumulation, and ultimately, leads to the death of ferritin in activated HSCs (Zhu et al., 2021a). In addition, N6-methyladenosine (m6A) modification is also associated with ferroptosis in liver HSCs, and FTO overexpression leads to the downregulation of m6A modification, which may damage autophagy and ferroptosis events. The m6A modification of BECN1 mRNA was significantly upregulated compared with other autophagy-related genes. YTHDF1 is a key reader protein for BECN1 mRNA stability, and the YTH domain extends the half-life of BECN1 mRNA, thereby activating autophagy and ultimately leading to ferroptosis in HSCs (Shen et al., 2021).

HSCs and hepatocytes play an important role in the physiological function of the liver, but few studies have reported the role of hepatocyte ferroptosis in the development of liver fibrosis. As liver parenchyma cells, liver cells have the largest number in the liver. Continuous hepatocyte injury and death can induce chronic inflammation and activation of HSCs, which is one of the initiations of the development of liver fibrosis (Gautheron et al., 2020). Iron overload is a driver of ferroptosis induction in hepatocytes, increasing the risk of developing liver fibrosis and cirrhosis. This excessive iron accumulation enhances ferroptosis in hepatocytes by inducing heme oxygenase-1 (HO-1) expression, which contributes to the progression of liver fibrosis, accompanied by the upregulation of FGF21 protein levels *in vitro* and *in vivo* (Wu et al., 2021). After iron overload induced ferroptosis, they release ROS and proinflammatory factors, transmit risk signals to surrounding cells including inflammatory cells and HSCs, and induce chronic inflammation and directly or indirectly induce the activation and proliferation of HSCs (Gautheron et al., 2020). Collagen synthesis after the activation of HSCs, ECM deposition, liver remodeling, and ultimately, fibrosis increased.

In the db/db mouse model of type 2 diabetes, the increased levels of TGF- β, collagen I, and collagen III in hepatocytes indicate a greater degree of hepatic fibrosis (Song et al., 2022). Ferroptosis activation was observed in hepatocytes; inhibition of GPX 4/GSH or impaired GSH synthesis increased ROS production (Yu et al., 2017); The Downregulation of SOD and upregulation of MDA, 4-HNE, and NOX4 increased the TfR1 expression, reduced the FPN 1 expression, and downregulated the SLC7A11 and Nrf 2/HO-1/ GPX4 signaling pathways (Song et al., 2022). Liraglutide suppresses hepatocyte ferroptosis and LF by increasing hepatic SLC7A11 and Nrf 2/HO-1/GPX 4 signaling expression. In addition, it reduces high glucose-induced LIP levels and intracellular lipid ROS levels *in vitro*, which is speculated to play a key role in reducing iron accumulation, oxidative damage, and ferroptosis (Song et al., 2022). Taken together, these findings suggest that the induction of ferroptosis in hepatocytes may be involved in the pathogenesis of liver fibrosis and can simultaneously promote liver fibrosis progression. However, the exact function of hepatocyte ferroptosis in the development and pathogenesis of liver fibrosis remains poorly understood. The molecular mechanisms regulating ferroptosis in hepatocytes remain largely unknown and require further investigation.

HSC activation is considered to be the core link of liver fibrosis, but it does not play an independent role in liver fibrosis and is regulated by the interaction network of hepatocytes and other non-parenchymal cells. It is crucial for the development and treatment of liver fibrosis to understand the difference of ferroptosis between HSCs and hepatocytes. Although these two cell classes play distinct roles in iron metabolism, their functions are tightly linked and interact. Therefore, further studies could reveal more detailed ferroptosis mechanisms between HSCs and hepatocytes, contributing to a comprehensive understanding of the pathway biology of liver disease and proposing more effective therapeutic strategies.

## 3 Mechanism and potential targets for targeting HSC ferroptosis to treat liver fibrosis

Antifibrotic treatments can be classified as drugs that mediate their antifibrotic effects through hepatocyte protection, inhibition of HSC activation (Bansal et al., 2019), and fibrotic scar evolution or immunomodulation (Schuppan et al., 2018; Bansal et al., 2019). However, despite numerous preclinical and clinical trials, no antifibrotic drugs have been approved by the Food and Drug Administration (FDA), and the only curative treatment option available for patients with advanced cirrhosis is liver transplantation (Neong et al., 2019). As the involvement of ferroptosis in the mechanism of LF has been studied, ferroptosis-based treatment of LF is a promising direction for future research. However, ferroptosis have both beneficial and detrimental effects on the progression of LF that need to be treated rationally. We can better understand the regulation of iron metabolism, lipid peroxidation, and oxidative stress by the inhibition of HSCs through ferroptosis.

### 3.1 Regulation of ferroptosis based on iron metabolism to intervene in liver fibrosis

The effects of disorders of iron metabolism are significant and can effectively influence the course of LF by regulating iron levels or by influencing the occurrence of ferritinophagy and, thus, regulating the outcome of cellular ferroptosis.

#### 3.1.1 Induction of ferroptosis regulates liver fibrosis

The role of ferritinophagy in LF has both advantages and disadvantages. On the one hand, it can lead to intracellular iron accumulation through ferritin autophagy, thereby reducing iron stores, causing an imbalance in intracellular iron homeostasis, triggering a signaling cascade to induce ferroptosis, causing the death of activated HSCs, and ultimately slowing down LF.

In recent years, several natural products and drugs have been effective in inducing ferroptosis by inducing ferritinophagy. Kong et al. (2019) found that artesunate could upregulate the expression of the vital ferritin phagocytosis marker LC3, which leads to ferritin autophagy in activated HSCs through the NCOA4-mediated autophagic pathway, thereby increasing the level of unstable iron in cells. The specific mechanism is the artesunate-induced co-localization of the ferroptosis marker GPX4, cyclooxygenase2 (Ptgs2) with the fibrosis marker α-SMA and the massive release of iron ions and excessive lipid peroxidation to the point of loss of antioxidant capacity, which in turn induces ferroptosis to attenuate LF. Zheng et al. (2022) showed that curcumol promotes autophagy in HSCs, mediates the degradation of NCOA4 and FTH 1 complexes to release iron ions, leading to iron overload, and induces ferroptosis to treat LF. Sui et al. (2018) reported that magnesium isoglycyrrhizinate reduced liver injury and LF scar formation by inducing ferroptosis in HSCs, and its mechanism of action was to promote iron enrichment and induce cellular ferroptosis by upregulating heme oxygenase-1 (HO-1) in HSCs and inhibiting its downstream target genes TF, TFR1, and FTH1. Wang et al. (2019) found that artemether (ART) induced ferroptosis in HSCs and did not affect normal liver cells. ART treatment increased the levels of iron, ROS, malondialdehyde (MDA), and 4-hydroxynonenal (4-HNE) and decreased the GSH and NADPH levels. ART-treated HSCs lead to cellular iron accumulation and ROS production, leading to cellular ferroptosis. ART can also occur through the p53-induced HSC ferroptosis. In addition, another study showed that the IRP 2-iron-ROS axis is required for ART. ART inhibits IRP2 ubiquitination and increases the iron content in HSCs, allowing IRP 2 to accumulate in HSCs and produce ROS, leading to the ferroptosis of cells (Li et al., 2020). Zhang et al. (2021) found that DHA was associated with ferroptosis and autophagy activation, and dihydroartemisinin (DHA) could inhibit the recombinant human platelet-derived growth factor-BB (PDGF-BB)-induced activation of HSCs. ATG5 siRNA and NCOA4 siRNA can inhibit DHA-mediated autophagy and ferroptosis, and ATG5 plasmid and NCOA4 plasmid can enhance the promoting effect of DHA effects on ferroptosis. Meanwhile, the N6-methyladenosine (m6A) modification is important for the activation of autophagy by DHA (Shen et al., 2022). DHA can inhibit the expression of demethylase FTO in HSCs, resulting in the increased level of m6A modification in HSCs. Furthermore, the level of m6A modification of BECN1 mRNA was significantly upregulated in DHA-induced HSC ferroptosis. The specific inhibition of m6A modification in HSCs can weaken DHA to induce HSC ferroptosis, so m6A can regulate the intervention mechanism of DHA (Shen et al., 2022). Zhang et al. (2018) found that ferroptosis induced by the ferroptosis inducers sorafenib and elastin-targeted induction of HSC ferroptosis, the RNA-binding protein embryonic lethal abnormal vision-like 1/human antigen R (ELAVL1/HuR) expression, can be significantly increased by inhibiting the ubiquitin-proteasome pathway, induce ferroptosis in HSCs, and relieve LF. Meanwhile, studies have shown that the inhibition of the activation of PERK signaling by GSK2656157 may enhance the sensitivity of HSCs to sorafini, thus enhancing the sorafini-induced ferroptosis of HSCs and subsequently alleviating liver fibrosis in mice (Jiang, 2022). Dilina and Armametty (Dilina and Armametty, 2023) found that the NaAsO2 caused the LX-2 iron ion level to increase to promote the occurrence of ferroptosis.

#### 3.1.2 Reducing iron overload in HSCs to slow down liver fibrosis

Activated HSCs are considered the major cellular source of ECM-secreting myofibroblasts for driving LF (Kowdley, 2004). Therefore, the inhibition of iron metabolism imbalance and excess iron accumulation in HSCs may slow down the development of LF. Yu et al. (2020) showed that using a hepatocyte Trf-LKO mouse model, a high-iron diet increased susceptibility to iron liver fibrosis. Their further treatment of Trf-LKO mice revealed that ferrostatin-1 rescued liver fibrosis. Furthermore, SLC39A14 was found to participate in intracellular Fe2 transport, implying that hepatic TRF deficiency can cause liver fibrosis. Huang et al. (2022) reported that isoliquiritigenin (ISL) relieved LF by inducing HSC ferroptosis through repressing GPX4 expression and increasing the expression of TFR and DMT1, thus producing a large number of ROS. Doxofylline (DOX) induced a significant increase in iron and ROS levels in HSCs, induced ferroptosis, and may be a promising anti-LF agent (Xu et al., 2023). Moreover, an RNA-binding protein, ZFP36/TTP (ZFP36 nomenclature protein), exhibits a mechanism to inhibit ferritinophagy. Erastin- and sorafenib-inducible ubiquitin ligase FBXW 7 and RSL3 reduced ZFP36 protein expression to construct a ZFP36 plasmid that can specifically bind to the (uuauuuuuuu) 3′-UTR autophagy-related 16 like 1 (ATG16L1) mRNA, increasing ATG16L1 instability and then inhibiting the activation of autophagy, resulting in reduced autophagic ferritin degradation, ultimately leading to ferroptosis (Zhang et al., 2020a). ZFP36 siRNA also inhibited ZFP 36 overexpression, thereby promoting iron death of HSCs. Zhang et al. (2020a) showed that the RNA-binding protein ZFP36 prevents ferroptosis by regulating the autophagic signaling pathway in HSCs. Li et al. (2022) revealed that the ellagic acid-induced reduction in plasma membrane FPN consistently resulted in intracellular ferritin accumulation and further increased ROS levels in activated HSCs, indicating that intracellular ROS is the ultimate cause of ferroptosis.

### 3.2 Regulation of ferroptosis based on lipid peroxidation intervenes in liver fibrosis

The currently identified mechanisms of ferroptosis regulation based on lipid peroxidation mainly target systemic lipid peroxidation and lead to lipid peroxidation by inhibiting GSH production and, thus, GPX4 activity and ROS accumulation (Yu et al., 2021). Zhu et al. (2021a) reported that TRIM26 was downregulated in fibrotic liver tissues and that the overexpression of TRIM26 accelerates the degradation of this protein by mediating SLC7A11 ubiquitination, promoting lipid peroxidation and, ultimately, leading to the death of activated HSC iron, suggesting that mediating SLC7A11 may promote HSC ferroptosis.

Du K et al. (2021) showed that inhibiting xCT/SLC7A11 could inhibit the process of HSC transdifferentiation and intracellular GSH synthesis, thus inducing HSC ferroptosis and reducing LF. Sorafenib-triggered HSC ferroptosis is accompanied by the reduction in SLC7A11 and HIF-1 α proteins. Furthermore, the investigators found that reduced HIF-1α and SLC7A11 in HSC-T6 cells led to sorafenib-induced cellular ferroptosis and reduced ECM. Conversely, increasing the expression of HIF-1α and SLC7A11 suppressed ferroptosis in HSCs and attenuates the antifibrotic effects of sorafenib (Yuan et al., 2022a). Lang et al., (2023) found that GRh 2 upregulates IRF1 expression, leading to the inhibition of SLC7A11, thus resulting in ferroptosis and inactivation of HSCs. GRh2 improves liver fibrosis by enhancing HSC ferroptosis and suppressing hepatic inflammation. Therefore, targeting the downregulation of SLC7A11-induced HSC ferroptosis may be a new way to treat LF.

However, there is a problem that we may overlook, that is, lipid peroxidation itself is one of the causative factors of LF, and too much emphasis is placed on inducing lipid peroxidation in HSCs, which leads to ferroptosis and then HSC death, to alleviate LF. This strategy is over the top, so some researchers have started investigating the inhibition of lipid peroxidation. It was demonstrated that tβ4 specifically binds to G-actin and regulates actin polymerization, thereby promoting vascular regeneration, wound healing, and hair follicle regeneration (Kim et al., 2016). Zhu et al. (2021b) found that Tβ4 protects hepatocytes by inhibiting the GPX4-mediated ferroptosis pathway and also protects hepatocytes by upregulating GPX4 expression to inhibit ferroptosis, thereby reducing oxidative stress and lipid peroxidation in the liver, which further reduces the recruitment of hepatic inflammatory factors and activation of apoptotic signaling pathways. Moreover, treatment with Fer-1, a ferroptosis inhibitor, increased the protective effect of Tβ4, while elastin, a ferroptosis inducer, attenuated the protective effect of Tβ4.

### 3.3 Subsection regulation of ferroptosis based on oxidative stress intervention in liver fibrosis

GPX4 is one of the critical targets in the regulation of ferroptosis. GPX 4 reduces hydroperoxidized phospholipids and fatty acids and protects against LPO-mediated oxidative stress, and inhibition of GPX 4 activity can accumulate through LPO at the cell membrane and induce cellular ferroptosis (Huang et al., 2023). Kuo et al. (2020) found that chrysophanol inhibited HSC activation, reduced SLC7A11, increased ROS levels, and promoted ferroptosis and endoplasmic reticulum stress activation, especially HBx-mediated inhibition of cell death through HBx-induced HSC activation of the GPX4 pathway and HPX4-independent pathway through GPX4 activation. Chrysophanol may exert an effect on activated HSCSs, induce ferroptosis, and prevent LF. Wild bitter melon (WM) treatment induces ROS accumulation and HSC death by enhancing endoplasmic reticulum stress and triggering ferroptosis in LPS-activated HSCs, suggesting that WM treatment has antifibrotic effects on LPS-activated HSCs (Ho et al., 2021). Luo et al. (2021) reported that celastrol could regulate the expression levels of GPX4 and COX-2 in different cells, suggesting its effects on the induction of ferroptosis. ISL promotes ferroptosis in HSCs by increasing MDA content and inhibiting GPX4 expression (Li et al., 2022). Decurin also could promote ferroptosis of HSCs by decreasing the levels of Gpx 4 and GSH (Que et al., 2022).

Yi et al. (2021) found that BBR causes autophagy inhibition in hematopoietic stem cells, enhances ROS and oxidative stress, promotes ferritin degradation, and increases the risk of redox-active iron accumulation and ROS increasing from the Fenton response. The Fenton response triggers lipid peroxidation and glutathione depletion, leading to ferroptosis and alleviating LF symptoms; in conclusion, BBR attenuates LF by inducing ferroredox-activated ROS-mediated ferroptosis of HSCs. Tan et al. (2022) recently revealed that human umbilical cord mesenchymal stem cells (hucMSCs) and their secreted exosomes (MSC-ex) can deliver BECN1 proteins to HSCs to increase their BECN1 expression and subsequently inhibit SLC7A11/xCT transcription in the nucleus. Reduced SLC7A11/xCT led to cysteine deficiency and reduced GSH in HSCs, which resulted in reduced GPX4 and the onset of ferroptosis.

### 3.4 Multitargeted regulation of ferroptosis to intervene in liver fibrosis

Based on the complex regulatory mechanisms of ferroptosis, multitargeted interventions are emerging as promising therapeutic strategies for LF. Exosomes are extracellular vesicles that contain proteins, DNA, and RNA of the cells that produce them and have also been found in recent years to have a vital role in the progression of LF (Dai et al., 2019; Kalluri et al., 2020). Transferrin receptor (TFRC) is a glycoprotein that can import iron, and silencing TFRC decreases total intracellular iron levels (Gammella et al., 2017). Exosomal miR-222 from HBV-infected hepatocytes promotes LF by inhibiting TFRC- and TFRC-induced HSC ferroptosis (Zhang et al., 2023).

Wogonoside (WG) improves LF through SOCS1/p53/SLC7A11 pathways to trigger the increase in SLC7A11, leading to the depletion of GPX 4 and GSH and an excess of ROS, finally inducing iron cell apoptosis in HSCs and inhibiting its activation during LF (Liu et al., 2022). Danshensu ameliorated LF by attenuating LPS-induced HSC activation by decreasing the expression of collagen І, CTGF, Gpx 4, SLC7A11, and LCN 2, and increasing the accumulation of lipid ROS (Wang et al., 2023). XCT-GSH-GPX 4 is an antioxidant signaling pathway. XCT consists of light-chain SLC7A11 and heavy-chain SCL3A2, which is a transmembrane amino acid transporter that participates in GSH synthesis through cystine uptake and glutamate excretion and promotes intracellular GSH synthesis (Yuan et al., 2022b). In acrylamide-induced infected cells, GPX4 and XCT expressions were decreased, and COX2 and GSH expressions were increased in HSC-T6 cells. Acrylamide-induced VDAC1 ensures mitochondrial integrity, regulated transport of substances and ions, and ROS production. This suggests that the induction of HSC ferroptosis by acrylamide is due to XCT-GSH-GPX4 antioxidant signaling and mitochondrial dysfunction (especially mtROS production) (Yuan et al., 2022b). Zhang et al. (2020b) found BRD7 upregulation, mitochondrial translocation of p53, a combination of SLC25A28 and p53, and ferroptosis induction in HSCs in patients with advanced hepatocellular carcinoma fibrosis treated with sorafenib. Concurrently, the HSC-specific blockade of the BRD7-p53-SLC25A28 axis abolished the erastin-induced HSC ferroptosis.

**FIGURE 2:**
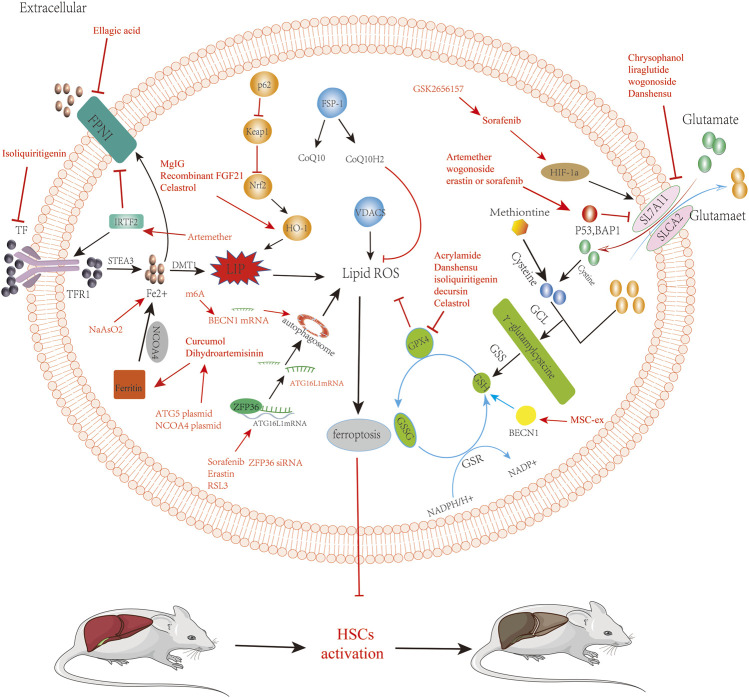
Reverse of liver fibrosis by inducing ferroptosis by bioactivated compounds in HSCs. Chrysophanol, liraglutide, wogonoside, danshensu relieve liver fibrosis by inhibiting SLC7A11 to induce ferroptosis. Artemether, wogonoside, erastin, or sorafenib relieve liver fibrosis by acting on p53 to induce ferroptosis, which is involved in regulating ferroptosis via systemic XC. Dihydroartemisinin and curcumol targeting acts on NCOA4, induces ferroptosis, and alleviates liver fibrosis. ATG5 plasmid and NCOA4 plasmid are able to promote the induction of iron death by dihydroartemisinin. Solafennib induced ferroptosis in HIF-1α through the HIF-1α/SLC7A11 pathway. GSK2656157 can promote solafennib to induce ferroptosis in HSCs. Acrylamide, danshensu, isoliquiritigenin, decursin, and celastrol inhibit GPX 4, thereby inducing ferroptosis in HSCs to alleviate liver fibrosis. Sorafenib, erastin, and RSL3 ZFP36 siRNA relieve liver fibrosis by acting on ZFP36 to induce ferroptosis. ATG16L1 mRNA can activate the autophagic ferritin, leading to the ferroptosis of HSCs. Artemether induced ferroptosis in HSCs to relieve liver fibrosis by upregulating IRTF2. Isoliquiritigenin induced ferroptosis in HSCs to relieve liver fibrosis by inhibiting TFR. Ellagic acid downregulates FPN 1 to induce ferroptosis and relieve liver fibrosis. BECN1 mRNA promotes the activation of autophagy to induce ferroptosis in HSCs. m6A induces ferroptosis in HSCs by stabilizing BECN1 mRNA to trigger autophagy activation. NaAsO2 can increase the level of iron ions to induce the iron death of HSCs.

As a new therapeutic modality, ferroptosis has been confirmed to play an important role in multiple systemic diseases. By targeting HSCs, we can induce the occurrence of ferroptosis. In treating LF, we summarize the current targets of ferroptosis in HSCs in Table 1 and drugs, small molecules target HSCs ferroptosis to treat liver fibrosis in Table 2. We describe the mechanism of targeting ferroptosis in HSCs in Figure 2. In the future, more mechanisms can be studied through HSCs, and relevant targeted drugs can be developed; therefore, targeting HSC ferroptosis is expected to be an important target for the treatment of LF, which is a key to inhibit iron metabolism, lipid peroxidation, and regulation of oxidative stress.

**TABLE 1:** Molecular signals involved in ferroptosis and liver fibrosis.

Target	Effect on HSC ferroptosis	Impact on LF	Mechanism	Disease model	Quotation
NCOA4	Restrain	Relieve	Releasing iron ions, cause iron overload	HSC-T6 cells	Zheng et al. (2022)
HO-1	Induction	Relieve	It promotes the accumulation of iron and lipid peroxides	CCL4-LF rat model	Sui et al. (2018)
p53	Induction	Relieve	P53-dependent ferroptosis	CCL4-LF mouse model	Wang et al. (2019)
IRP2	Induction	Relieve	Promoting iron overload	CCL4-LF mouse model	Li et al. (2020)
NCOA4	Induction	Relieve	Activation of autophagy and upregulation of NCOA4	CCL4-LF rats model	Zhang et al. (2021)
ELAVL1	Induction	Relieve	It promotes autophagic ferritin degradation	CCL4-LF mouse model	Zhang et al., 2018
GPX4	Restrain	Relieve	GPX 4 was downregulated and COX-2 was upregulated	C57BL/6 male mice	Luo et al., 2022
HO-1	Induction	Relieve	Upregulates HO-1 to induce ferroptosis and alleviate liver fibrosis	LX-2 cell	Luo et al. (2022)
GPX4	Restrain	Relieve	Inhibiting GPX4	CCL4-LF mouse model	Huang et al. (2022)
TFR	Induction	Relieve	Adding TFR and DMT1	HSC-T6	Huang et al. (2022)
HO-1	Induction	Relieve	Inhibition of HO-1 and activation of Nrf2	Iron overload-induced mouse model	Wu et al. (2021)
ZFP36	Induction	Relieve	Downregulaton of ZFP36, activation of phagocytoferrin, and induction of ferroptosis	BDL-LF mouse model	Zhang et al. (2020a)
FPN	Induction	Relieve	Inducing FPN-dependent ferroptosis in HSC	CCL-induced male C57BL/6 mice	Li et al. (2022)
HIF-1α	Induction	Relieve	Ferroptosis was induced through the HIF-1α/SLC7A11 pathway	CCL4-LF mouse model	Yuan et al. (2022a)
SLC7A11	Induction	Relieve	Accumulation of lipid reactive oxygen species	NA	Kuo et al. (2020)
BECN1	Restrain	Relieve	BECN1 delivery promotes xCT/GPX4-mediated ferroptosis of hematopoietic stem cells	CCL4-LF mouse model	Tan et al. (2022)
SLC7A11	Restrain	Relieve	Inhibition of ferroptosis by increasing the expression of SLC7A11 and the Nrf2/HO-1/GPX4 signaling pathway	Diabetic mouse model	Song et al. (2022)
BRD7-p53-SLC25A28 axis	Induction	Relieve	It promotes p53 mitochondrial translocation	BDL-LF mouse model	Zhang et al. (2020b)
SOCS1/p53/SLC7A11	Restrain	Relieve	Consumption of SLC7A11, GPX4, and GSH and accumulation of iron, ROS, and MDA	HSC-T6 cells	Liu et al. (2022)
Gpx4,SLC7A11	Restrain	Relieve	Dan attenuates LPS-induced HSC activation during liver fibrosis by regulating ferroptosis in LX-2 and T6 cells	LX-2 and T6 cells	Wang et al. (2023)
XCT-GSH-GPX4	Induction	Relieve	Affecting XCT-GSH-GPX 4 antioxidant signaling	HSC-T6 cells	Yuan et al. (2022b)

HO-1, heme oxygenase-1; CCl4-LF, carbon tetrachloride-induced liver fibrosis; BDL-LF, bile duct ligation-induced liver fibrosis; MCD-NASH, methionine- and choline-deficient feed-feeding-induced non-alcoholic steatohepatitis; HFD-NAFLD, high-fat diet-induced non-alcoholic fatty liver disease; IRP2, iron regulatory protein 2; NCOA4, nuclear receptor coactivator protein 4; ELAVL1, ELAV-like protein 1; m6A, N6-methyladenosine; FGF21, fibroblast growth factor 21; ZFP36, human zinc finger protein 36; TRIM26, triplex motif-containing 26; xCT, cystine/glutamate reverse transport system; SLC7A11, solute carrier family 7 member 11; HIF-1α, hypoxia-inducible factor 1α; ENO3, enolase 3; BRD7, bromodomain-containing protein 7.

**TABLE 2:** Drugs and small molecules target HSCs ferroptosis to alleviate liver fibrosis.

Drugs	Target	Effect on HSC ferroptosis	Mechanism	Disease model	Quotation
Artesunate	NA	Induction	Ferritin phagocytosis is triggered	CCL4-LF mouse model	Kong et al. (2019)
Curcumol	NCOA4	Induction	Releasing iron ions and causing iron overload	HSC-T6 cells	Zheng et al. (2022)
Magnesium isoglycyrrhizinate	HO-1	Induction	It promotes the accumulation of iron and lipid peroxides	CCL4-LF rats model	Sui et al. (2018)
Artemether	p53	Induction	P53-dependent ferroptosis	CCL4-LF mouse model	Wang et al. (2019)
Artemether	IRP2	Induction	Promotes iron overload	CCL4-LF mouse model	Li et al. (2020)
Dihydroartemisinin	NCOA4	Induction	Activation of autophagy and the upregulation of NCOA4	CCL4-LF rat model	Zhang et al. (2021)
Sorafenib	ELAVL1	Induction	It promotes autophagic ferritin degradation	CCL4-LF mouse model	Zhang et al. (2018)
Celastrol	GPX4	Induction	GPX 4 was downregulated and COX-2 was upregulated	C57BL/6 male mice	Luo et al. (2022)
Celastrol	HO-1	Induction	Upregulating HO-1 to induce ferroptosis and alleviate liver fibrosis	LX-2 cell	Luo et al. (2022)
Isoliquiritigenin	GPX4	Induction	Inhibiting GPX4	CCL4-LF mouse model	Huang et al. (2022)
Isoliquiritigenin	TFR	Induction	Adding TFR and DMT1	HSC-T6	Huang et al. (2022)
Doxofylline	NA	Induction	Increase in iron and ROS levels in HSCs	CCL4-LF mouse model	Xu et al. (2023)
Recombinant FGF21	HO-1	Induction	Inhibition of HO-1 and activation of Nrf2	Iron overload-induced mouse model	Wu et al. (2021)
Sorafenib, erastin, or RSL3	ZFP36	Induction	Downregulation of ZFP36, activation of phagocytoferrin, and induction of ferroptosis	BDL-LF mouse model	Zhang et al. (2020a)
Ellagic acid	FPN	Induction	Inducing FPN-dependent ferroptosis in HSCs	CCL-induced male C57BL/6 mice	Li et al. (2022)
Sorafenib	HIF-1α	Induction	Ferroptosis was induced through the HIF-1α/SLC7A11 pathway	CCL4-LF mouse model	Yuan et al. (2022a)
Chrysophanol	SLC7A11	Induction	Accumulation of lipid reactive oxygen species	NA	Kuo et al. (2020)
Wild bitter melon extract	NA	Induction	Reactive oxygen species accumulation	NA	Ho et al. (2021)
Berberine	NA	Induction	Autophagic lysosomal pathway is impaired, and cellular ROS production is increased	CCL4-LF mouse model/TAA-LF mouse model	Tan et al. (2022)
MSC-ex	BECN1	Induction	BECN1 delivery promotes xCT/GPX4-mediated ferroptosis of HSCs	CCL4-LF mouse model	Tan et al. (2022)
Erastin or sorafenib	BRD7-p53-SLC25A28 axis	Induction	It promote sp53 mitochondrial translocation	BDL-LF mouse model	Zhang et al. (2020b)
Wogonoside	SOCS1/P53/SLC7A11	Induction	Consumption of SLC7A11, GPX4, and GSH and accumulation of iron, ROS, and MDA	HSC-T6 cells	Liu et al. (2022)
Decursin	GPX4	Induction	Decursin promoted ferroptosis in activated HSCs *in vitro* by declining Gpx4 and GSH levels	CCL4-LF mouse model	Que et al. (2022)
Danshensu	Gpx4,SLC7A11	Induction	Dan attenuates LPS-induced HSC activation during liver fibrosis by regulating ferroptosis in LX-2 and T6 cells	LX-2 and T6 cells	Wang et al. (2023)
Acrylamide	XCT-GSH-GPX4	Induction	AA induces episodes of ferroptosis by affecting XCT-GSH-GPX4 antioxidant signaling	HSC-T6 cells	Yuan et al. (2022b)
BECN1 mRNA	NA	Induction	Activation of autophagy	LX-2 and T6 cells	Shen et al. (2021)
m6A	BECN1 mRNA	induction	Triggering autophagy activation by stabilizing BECN1 mRNA	LX-2 and T6 cells	Shen et al. (2021)
ATG5 plasmid and NCOA4 plasmid	NA	Induction	Promoting effects of DHA effects on HSC ferroptosis	CCL4-LF rat model	Zhang et al. (2021)
GSK2656157	NA	Induction	Enhancing the sensitivity of HSCs to sorafini	CCL4-LF rat model	Jiang (2022)
NaAsO2	NA	Induction	Elevated iron ion levels	LX-2 cell	Dilina and Armametty, 2023
ZFP36 siRNA	NA	Induction	Suphibiting the overexpression of ZFP36, triggering autophagy activation, leading to ferroptosis in HSCs	HSC-T6 cells	Zhang et al. (2020a)
ATG16L1 mRNA	NA	Induction	Activation of autophagic ferritin, leading to ferroptosis of HSCs	HSC-T6 cells	Zhang et al. (2020a)
GRh 2	IRF1	Induction	Leading to the inhibition of SLC7A11, thus resulting in HSC ferroptosis	CCL4-LF rats model	Lang et al. (2023)

HO-1, heme oxygenase-1; CCl4-LF, carbon tetrachloride-induced liver fibrosis; IRP2, iron regulatory protein 2; BDL-LF, bile duct ligation-induced liver fibrosis; HIF-1α, hypoxia-inducible factor 1α; SLC7A11, solute carrier family 7 member 11; IRP2, iron regulatory protein 2; NCOA4, nuclear receptor-assisted activator protein 4; ELAVL1, ELAV-like protein 1; ZFP36, human zinc finger protein 36; RSL3, GSH, peroxidase 4 inhibitor; BECN1, benzyl chloride 1; TAA-LF, thioacetamide-induced liver fibrosis; SLC25A28, solute carrier family 25 member 28; NA, not applicable.

The specific mechanism of LF ferroptosis has not been fully clarified; more detailed signal transduction pathway is still in further exploration. Many studies are still in the cell or animal experimental stage because of the lack of large samples, multicenter clinical randomized controlled studies, and evidence; we still need to conduct a more comprehensive and in-depth study to further explore the relationship between ferroptosis and LF. For targeting HSCs to treat LF, there is still a transformation gap; therefore, researchers should convert putative targets into effective clinical drugs. For disease treatment based on ferroptosis, induced ferroptosis may have the dual effect of injury and treatment, and according to the disease background, related treatment is needed to limit the occurrence of side effects. For the occurrence of ferroptosis mechanism development, new targeted drugs to regulate the occurrence of cell ferroptosis in different disease types is expected to become the new trend of disease treatment in the future.

## 4 Summary and outlook

Since the concept of ferroptosis was introduced, it has become the most sought-after form of regulated cell death. In this review, we summarize recent studies based on iron metabolism, lipid peroxidation, and oxidative stress that regulate the occurrence of ferroptosis and, thus, affect the development and progression of LF. Although more and more studies have been reported, the molecular mechanisms and signaling pathways of ferroptosis in the process of LF are not very clear. For example, (1) current studies suggest that elevated levels of iron ions and lipid peroxidation are necessary for the occurrence of ferroptosis, but there is evidence that elevated levels of iron ions and lipid peroxidation are not at all beneficial to the progression of LF, as they may have some impact on other normal hepatocytes and the intrahepatic cellular environment. All current studies on regulating LF progression by ferroptosis have escaped the classical model of ferroptosis, namely, the traditional GPX4/ACSL4 model (Doll et al., 2017). In recent years, Liu et al. (2022) identified a new mechanism of ferroptosis mediated by p53 that is different from the classical model of ferroptosis. However, this research has yet to be integrated into the field of LF. We do not yet know the specific effect of ferroptosis induced by the new mechanism on LF. It remains to be explored whether the new mechanism could be used to find a way to target ferroptosis and remove activated HSCs without affecting other normal hepatocytes. (2) Unlike ferroptosis treatment for hepatocellular carcinoma, which has long been used in clinical practice, clinical studies targeting ferroptosis for LF have not yet been reported. We hope that someone will shed more light on the specific mechanisms of ferroptosis execution in the future. A fundamental question is how to induce ferroptosis in cells, which is the key to the final clinical application of targeted ferroptosis therapy for LF. (3) There are no specific markers of ferroptosis in the organism, and specific reference indicators can be found in the future, which could provide a basis for future clinical diagnosis. In conclusion, continuous exploration of the ferroptosis mechanism and targeting ferroptosis may be a promising treatment strategy for LF and provide a good reference for treating ferroptosis in other diseases.
